# Psychological distress among health professional students during the COVID-19 outbreak

**DOI:** 10.1017/S0033291720001555

**Published:** 2020-05-11

**Authors:** Yuchen Li, Yue Wang, Jingwen Jiang, Unnur A. Valdimarsdóttir, Katja Fall, Fang Fang, Huan Song, Donghao Lu, Wei Zhang

**Affiliations:** 1 Mental Health Center, West China Hospital of Sichuan University, Chengdu, China; 2 Department of Medical Epidemiology and Biostatistics, Karolinska Institutet, Stockholm, Sweden; 3 West China Biomedical Big Data Center, West China Hospital, Sichuan University, Chengdu, China; 4 Center of Public Health Sciences, Faculty of Medicine, University of Iceland, Reykjavík, Iceland; 5 Department of Epidemiology, Harvard T.H. Chan School of Public Health, Boston, Massachusetts, USA; 6 Clinical Epidemiology and Biostatistics, School of Medical Sciences, Örebro University, Örebro, Sweden; 7 Institute of Environmental Medicine, Karolinska Institutet, Stockholm, Sweden; 8 Clinical Research Center for Breast Diseases, West China Hospital, Sichuan University, Chengdu, China; 9 Channing Division of Network Medicine, Brigham and Women's Hospital, Harvard Medical School, Boston, Massachusetts, USA

**Keywords:** Psychological distress, health professional students, COVID-19, acute stress reaction, trauma

## Abstract

**Background:**

Due to the drastic surge of COVID-19 patients, many countries are considering or already graduating health professional students early to aid professional resources. We aimed to assess outbreak-related psychological distress and symptoms of acute stress reaction (ASR) in health professional students and to characterize individuals with potential need for interventions.

**Methods:**

We conducted a prospective cohort study of 1442 health professional students at Sichuan University, China. At baseline (October 2019), participants were assessed for childhood adversity, stressful life events, internet addiction, and family functioning. Using multivariable logistic regression, we examined associations of the above exposures with subsequent psychological distress and ASR in response to the outbreak.

**Results:**

Three hundred and eighty-four (26.63%) participants demonstrated clinically significant psychological distress, while 160 (11.10%) met the criterion for a probable ASR. Individuals who scored high on both childhood adversity and stressful life event experiences during the past year were at increased risks of both distress (ORs 2.00–2.66) and probable ASR (ORs 2.23–3.10), respectively. Moreover, internet addiction was associated with elevated risks of distress (OR 2.05, 95% CI 1.60–2.64) and probable ASR (OR 2.15, 95% CI 1.50–3.10). By contrast, good family functioning was associated with decreased risks of distress (OR 0.43, 95% CI 0.33–0.55) and probable ASR (OR 0.48, 95% CI 0.33–0.69). All associations were independent of baseline psychological distress.

**Conclusions:**

Our findings suggest that COVID-19 related psychological distress and high symptoms burden of ASR are common among health professional students. Extended family and professional support should be considered for vulnerable individuals during these unprecedented times.

## Introduction

In the wake of the sudden 2019 novel coronavirus disease (COVID-19) pandemic, healthcare workers are not only at risk for physical challenges but also mental burden, particularly psychological distress (Lai et al., 2020; Liu et al., 2020). Due to the drastic surge of patients worldwide, many countries are considering or already graduating senior health professional students early to join the workforce. Meanwhile, inexperience in such urgent situations may be particularly stressful for students graduating directly to responsibilities in the pandemic crisis. Leveraging a prospective cohort of health professional students in China, we aimed to assess the COVID-19 related psychological distress and to identify high-risk groups who might benefit from supportive measures.

## Methods

The Health Professional Students' Health is an ongoing prospective cohort investigating psychosocial wellbeing in health professional students at Sichuan University, China. At baseline (October 2019), about 82% of students (*n* = 2025) were enrolled and completed assessment for childhood adversity, stressful life event experiences, internet addiction, and family functioning.

During February 7–13, 2020, we invited all participants for a special assessment of the COVID-19 related mental health and 1442 (71.2%) completed (764 medical, 211 nursing, and 467 medical technology students). We evaluated psychological distress using the Kessler 6-item Psychological Distress Scale (K6) and acute stress reaction (ASR) using the Impact of Event Scale-Revised (IES-R). The questions were phrased specific to the outbreak. Using validated cutoffs, ⩾5 points in K6 was classified as having significant psychological distress, while ⩾24 points in IES-R as probable ASR. All assessments were conducted using web-based, validated questionnaires (see online Supplementary Table S1).

We estimated odds ratios of distress and ASR by contrasting individuals of high *v.* low level of baseline psychosocial factors using logistic regression with adjustment for demographic and socioeconomic factors obtained from the baseline. To evaluate the impact of pre-pandemic distress, we additionally adjusted for baseline depression, anxiety, and stress as a composite measure of distress assessed by the Depression, Anxiety and Stress Scale-21 item. This study was approved by the Ethics Committee of the Sichuan University and electronic consent forms were obtained from all participants.

## Results

Among the 1442 students, 200 (13.87%) had started their internship (usually the last year in medical school) and 33 (2.29%) were quarantined inside the Hubei Province (the epicenter at the time). None reported COVID-19, while 13 (0.90%) had at least one relative infected. About two weeks after the nationwide quarantine, 384 (26.63%) students reported clinically significant psychological distress, while 160 (11.10%) met criteria for a probable ASR, in response to the COVID-19 outbreak. A total of 131 (9.08%) students showed signs of both distress and ASR. Students who showed psychological distress were more likely to be female and at higher levels of baseline depression, anxiety, and stress, compared with those who did not show distress (*p* < 0.05; online Supplementary Table S2). Other demographic, lifestyle, and socioeconomic factors were largely similar between the groups. A similar pattern was found for ASR.

Students who scored high on childhood adverse experiences were at increased risks of distress (OR 2.00, 95% CI 1.55–2.58) and ASR (OR 2.23, 95% CI 1.56–3.23), respectively (Table 1). Similar patterns, yet even with higher magnitude associations were found with previous stressful life events. Moreover, internet addiction was associated with elevated risks of distress (OR 2.05, 95% CI 1.60–2.64) and probable ASR (OR 2.15, 95% CI 1.50–3.10). In contrast, good family functioning was associated with decreased risks of distress (OR 0.43, 95% CI 0.33–0.55) and probable ASR (OR 0.48, 95% CI 0.33–0.69). All associations were independent of baseline psychological distress, despite attenuated effect size. Similar patterns were observed for distress and ASR symptoms.

**Table 1.** Factors associated with psychological distress during the COVID-19 outbreak in health professional students: a prospective cohort study in China

	Psychological distress ^a^			ASR^b^		
	Cases	OR (95% CI) ^c^	OR (95% CI) ^d^	Cases	OR (95% CI) ^c^	OR (95% CI) ^d^
Childhood adverse experiences ^e^						
Low (*n* = 720)	151	Ref.	Ref.	56	Ref.	Ref.
High (*n* = 691)	222	2.00 (1.55–2.58)	1.44 (1.10–1.89)	101	2.23 (1.56–3.23)	1.64 (1.11–2.41)
Stressful life-event experiences^f^						
Low (*n* = 700)	126	Ref.	Ref.	44	Ref.	Ref.
High (*n* = 699)	242	2.66 (2.06–3.46)	1.70 (1.28–2.27)	108	3.10 (2.13–4.59)	2.30 (1.51–3.52)
Internet addiction^g^						
No (*n* = 720)	141	Ref.	Ref.	53	Ref.	Ref.
Yes (*n* = 671)	225	2.05 (1.60–2.64)	1.44 (1.10–1.89)	100	2.15 (1.50–3.10)	1.57 (1.07–2.32)
Family functioning^h^						
Low (*n* = 724)	237	Ref.	Ref.	101	Ref.	Ref.
High (*n* = 659)	126	0.43 (0.33–0.55)	0.60 (0.45–0.79)	51	0.48 (0.33–0.69)	0.67 (0.45–1.00)

CI, confidence interval; IES-R, The Impact of Event Scale-Revised; K6, Kessler 6-item Psychological Distress Scale; N, number; OR, odds ratio; Ref., reference.

a Psychological distress using the Kessler 6-item Psychological Distress Scale (K6).

b ASR using the impact of event scale-revised (IES-R).

c The estimates were adjusted for age, sex, BMI, training program, family background, paternal and maternal occupations, paternal and maternal educational levels, being the only child, and being the left-behind child.

d The estimates were additionally adjusted for baseline depression, anxiety, and stress level.

e In this analysis, 31 (2.15%) individuals who missed the measure of childhood adverse experiences were not included.

f In this analysis, 43 (2.98%) individuals who missed the measure of stressful life event experiences were not included.

g In this analysis, 51 (3.54%) individuals who missed the measure of internet addiction were not included.

At the time of the survey, students were most concerned about having COVID-19 (67.51%; see online Supplementary Table S3). If they were to work during the outbreak, personal protective equipment was stated as most important (92.72%). About one fifth of the students (19.14%) reported that the pandemic would affect their future career choice; among them, only a half (56.03%) stated they would practice health professional after graduation. Such proportion was 10% higher among individuals who answered, ‘career choice not being affected’ (61.44%).

## Discussion

To the best of our knowledge, this is the first study showing that the COVID-19 outbreak-related psychological distress and ASR symptoms are common in uninfected health professional students in China. More importantly, using prospective data, we illustrated that good family functioning is associated with decreased risks, whereas internet addiction and childhood and recent adverse experiences are associated with a greater burden of distress and ASR during the COVID-19 outbreak, independent of pre-pandemic distress.

Our data show that about 27% of health professional students experienced significant distress during the outbreak, while 11% had probable ASR which may develop into PTS disorder if not correctly addressed (Soldatos, Paparrigopoulos Tj Fau – Pappa, Pappa Da Fau – Christodoulou, & Christodoulou, 2006). Although symptoms are common, the prevalence is much lower in students, at least in the early phase of the outbreak, than that in healthcare workers. Lai et al. reported that about 72% of healthcare workers had probable ASR assessed by the same questionnaire, although 60% of the participants were working frontline in Wuhan (Lai et al., 2020), the first epicenter. The notable difference in prevalence between health professional students and frontline workers may signal a potentially critical development if inexperienced students are graduating into this battle without support. It is, however, noteworthy that we only assessed distress once in the early phase of the outbreak. Given the ongoing pandemic, further studies on mental health over time and a confirmation of ASR development after trauma are needed.

It is well-documented that family or social support after trauma has a protective effect on PTS symptoms (Tortella-Feliu et al., 2019). These data add to the knowledge that emotional support from the family before trauma may better prepare an individual's resilience to stress, independently of baseline stress levels. Social media use is often correlated with anxiety (Vannucci, Flannery, & Ohannessian, 2017). Our prospective data showed that students reported some signs of internet addiction are at higher risk of distress and ASR during the outbreak. Future studies are needed to assess whether expanding online consulting particularly during the outbreak is effective to reduce stress among individuals with internet addiction. It is well-known that adverse experiences are associated with higher stress levels (Tortella-Feliu et al., 2019). Our findings confirmed the role of adversities in response to the outbreak.

An important limitation of this study is that, due to the use of different questionnaires over time, we were not able to estimate an interpretable change of distress from baseline to the outbreak. We showed, however, that about 22% of students with low stress levels at baseline developed significant outbreak-related distress, which may shed light on the mental burden imposed by this crisis. Second, around 30% of cohort enrollees did not participate in the COVID-19 survey, although the baseline characteristics (except a higher proportion of males and senior/medical students) including levels of psychological distress were largely comparable (data not shown).

## Conclusions

Our findings suggest that symptoms of psychological distress and ASRs are common among health professional students during the COVID-19 outbreak. As such symptoms may negatively affect job performance of healthcare workers (Kolehmainen et al., 2015) and their health if persisting (Bisson, 2019), assessment of distress may be implemented if and when the students are accelerating to the frontline. Our findings may provide first-hand evidence to help identify those who might benefit the most from family and psychological support during and after these unprecedented times.

## Supplementary material

For supplementary material accompanying this paper visit http://dx.doi.org/10.1017/S0033291720001555. click here to view supplementary material
