# Psychological distress among health professional students during the COVID-19 outbreak – Corrigendum

**DOI:** 10.1017/S0033291721000714

**Published:** 2021-08

**Authors:** Yuchen Li, Yue Wang, Jingwen Jiang, Unnur A. Valdimarsdóttir, Katja Fall, Fang Fang, Huan Song, Donghao Lu, Wei Zhang

**Affiliations:** 1 Mental Health Center, West China Hospistal of Sichuan University, Chengdu, China; 2 West China Biomedical Big Data Center, West China Hospital, Sichuan University, Chengdu, China; 3 Department of Medical Epidemiology and Biostatistics, Karolinska Institutet, Stockholm, Sweden; 4 Center of Public Health Sciences, Faculty of Medicine, University of Iceland, Reykjavík, Iceland; 5 Department of Epidemiology, Harvard T.H. Chan School of Public Health, Boston, Massachusetts, USA; 6 Clinical Epidemiology and Biostatistics, School of Medical Sciences, Örebro University, Örebro, Sweden; 7 Institute of Environmental Medicine, Karolinska Institutet, Stockholm, Sweden; 8 Clinical Research Center for Breast Diseases, West China Hospital, Sichuan University, Chengdu, China; 9 Channing Division of Network Medicine, Brigham and Women's Hospital, Harvard Medical School, Boston, Massachusetts, USA

This article was published in Psychological Medicine with an error in the author's affiliations. This has now been corrected online and in the article.

The authors apologise for this error.
