# Supplementary material for: Psychological distress among health professional students during the COVID-19 outbreak
Source: Psychol Med. 2020 May 11:1–3. doi: 10.1017/S0033291720001555 (PMC7225209; doi:10.1017/S0033291720001555)
Supplement: Supplementary file 1 [file S0033291720001555sup001.docx]

**Supplementary Materials**

**S1. Supplementary Methods**

**S2. Supplementary Tables**

**S3. Supplementary Tables**

**S1. Supplementary Methods**

**S1A. Assessment of baseline psychosocial factors**

At baseline, participants were assessed for psychosocial factors as follows.

1) Childhood adversity: The Childhood Trauma Questionnaire-Short Form is a 28-item self-report scale evaluating traumatic experiences before age of 16 years(He, Zhong, Gao, Xiong, & Yao, 2019). The total score ranges from 25 to 125 and was classified into low and high by the median. The scale was proved of adequate internal consistency, with the Cronbach’s alpha of 0.872.

2) Stressful life events: As the average age was < 20 years in our sample, the Adolescent Self-Rating Life Events Check-List(Xiu-Hong & Shu-Qiao, 2015) with 27 items was used to measure the frequency and intensity of stressful life events commonly experienced during the past year. The total score ranges from 0 to 135 and was classified into low and high by the median. The Cronbach’s alpha was 0.928.

3) Internet addiction: The Internet Addiction Test is a 20-item measure of compulsive use of the internet in adults and adolescents(Netaddiction.com, 2018). The total score ranges from 0 to 100 and we used ≥31 to indicate the presence of internet addiction. The Cronbach’s alpha was 0.942.

4) Family functioning: The general functioning subscale of the Family Assessment Device is a 12-item measure of overall family functioning(Chen et al., 2003). The total score ranges from 0 to 4 and was classified into low and high by the median. The Cronbach’s alpha was 0.915.

**S1B. Assessment of COVID-19 related mental health**

During the outbreak, we specifically assessed psychological distress and symptoms of acute stress reaction (ASR) in response to the COVID-19 crisis. Although the University is located in the Sichuan Province, the 11^th^ most-hit province in China at the time of the survey, most students were at home across the entire country.

1) Psychological distress is assessed using Kessler 6-item Psychological Distress Scale, a 6-item measure rated on a 5-point Likert scale which primarily assesses depression- and anxiety-related symptoms during the past 30 days. The total score ranges from 0 to 24 and ≥5 was classified as having significant psychological distress according to a validation study in the Asian population^(Furukawa et al., 2008)^. The Cronbach’s alpha was 0.909.

2) Symptoms of ASR represent the presence of posttraumatic stress (PTS) symptoms shortly after a trauma or distressing event. The Impact of Event Scale-Revised is a 22-item self-report measure of PTS symptoms including intrusion, avoidance, and hyperarousal symptoms during the past seven days using a 5-point Likert scale. The total score ranges from 0 to 88 and ≥24 has been successfully used to identify ASR(Imsiragic, Begic, & Martic-Biocina, 2009; Wu & Chan, 2003). The Cronbach’s alpha was 0.914.

Additionally, we included several multiple-choice questions about the student’s concerns about the outbreak and their needs if they were to practice during the outbreak. We also surveyed whether and how the outbreak affected their career choice for the future.

**S1C. Covariates**

We obtained information on age, sex, admission year, training program, smoking, body mass index (BMI), family background, being the only child, and being a “left-behind child” (i.e., individuals were taken care of by their extended families in childhood while parents left to work in urban areas) from the baseline survey. We also extracted parental educational levels and occupations as proxies for socioeconomic status. Baseline psychological distress, including depression/anxiety symptoms and stress level, was measured using the Depression, Anxiety and Stress Scale-21 item (DASS-21)(Lovibond, Lovibond, & Australia, 1995). The cut-off values for significant depression/anxiety symptoms and stress level were 10, 8, and 15, respectively. The Cronbach’s alpha was 0.925.

**S1D. References**

Chen, J.-L., Kennedy, C., Kools, S., Slaughter, R. E., Franck, L., Kong, S. K., & Wong, T. K. (2003). Culturally appropriate family assessment: analysis of the Family Assessment Device in a pediatric Chinese population. *Journal of nursing measurement, 11*(1), 41-60. doi:10.1891/106137403780954994

Furukawa, T. A., Kawakami, N., Saitoh, M., Ono, Y., Nakane, Y., Nakamura, Y., . . . Kikkawa, T. (2008). The performance of the Japanese version of the K6 and K10 in the World Mental Health Survey Japan. *International Journal of Methods in Psychiatric Research, 17*(3), 152-158. doi:10.1002/mpr.257

He, J. Y., Zhong, X., Gao, Y. D., Xiong, G., & Yao, S. Q. (2019). Psychometric properties of the Chinese version of the Childhood Trauma Questionnaire-Short Form (CTQ-SF) among undergraduates and depressive patients. *Child Abuse & Neglect, 91*, 102-108. doi:10.1016/j.chiabu.2019.03.009

Imsiragic, A. S., Begic, D., & Martic-Biocina, S. (2009). Acute Stress and Depression 3 Days after Vaginal Delivery - Observational, Comparative Study. *Collegium antropologicum, 33*(2), 521-527. Retrieved from <Go to ISI>://WOS:000268067400025

Lovibond, S. H., Lovibond, P. F., & Australia, P. F. o. (1995). Manual for the depression anxiety stress scales (2nd ed). *Psychology Foundation of Australia, Sydney, N.S.W*.

Netaddiction.com. (2018). Internet Addiction Test (IAT) Manual. Retrieved from http://cyberpsy.ru/wp-content/uploads/2018/02/iat-manual.pdf.

Wu, K. K., & Chan, K. S. (2003). The development of the Chinese version of Impact of Event Scale - Revised (CIES-R). *Social Psychiatry and Psychiatric Epidemiology, 38*(2), 94-98. doi:10.1007/s00127-003-0611-x

Xiu-Hong, X., & Shu-Qiao, Y. (2015). Validity and reliability of the Adolescent Self-rating Life Events Checklist in middle school students [in Chinese]. *Chinese Mental Health Journal, 29*(05), 355-360. Retrieved from <http://www.cnki.com.cn/Article/CJFDTOTAL-ZXWS201505012.htm>

**S2. Supplementary Tables**

**Table S2. Baseline characteristics and psychological distress during the COVID-19 outbreak in health professional students: a prospective cohort study in China – mean ± SD or N (%).**

|  | Psychological distress ^a^ | |  | Acute stress reaction ^b^ | |  |
| --- | --- | --- | --- | --- | --- | --- |
|  | No (N=1058) | Yes (N=384) | P | No (N=1282) | Yes (N=160) | P |
| Age, years | 19.99±1.51 | 20.02±1.55 | 0.81 | 20.00±1.51 | 19.98±1.62 | 0.873 |
| Sex |  |  |  |  |  |  |
| Female | 634 (59.92) | 257 (66.93) | 0.02 | 777 (60.61) | 114 (71.25) | 0.01 |
| Male | 424 (40.08) | 127 (33.07) |  | 505 (39.39) | 46 (28.75) |  |
| Admission year |  |  |  |  |  |  |
| 2015 | 69 (6.53) | 24 (6.25) | 0.50 | 86 (6.71) | 7 (4.38) | 0.43 |
| 2016 | 168 (15.88) | 65 (16.93) |  | 205 (15.99) | 28 (17.50) |  |
| 2017 | 281 (26.65) | 89 (23.18) |  | 329 (25.66) | 41 (25.62) |  |
| 2018 | 233 (22.02) | 99 (25.78) |  | 288 (22.46) | 44 (27.50) |  |
| 2019 | 307 (29.02) | 107 (27.86) |  | 374 (29.17) | 40 (25.00) |  |
| Program |  |  |  |  |  |  |
| Medicine | 574 (54.25) | 190 (49.48) | 0.22 | 692 (53.98) | 72 (45.00) | 0.10 |
| Nursing | 147 (13.89) | 64 (16.67) |  | 183 (14.27) | 28 (17.50) |  |
| Medical technology | 337 (31.85) | 130 (33.85) |  | 407 (31.75) | 60 (37.50) |  |
| Smoking |  |  |  |  |  |  |
| No | 1038 (98.11) | 378 (98.44) | 0.85 | 1258 (98.13) | 158 (98.75) | 0.81 |
| Yes | 20 (1.89) | 6 (1.56) |  | 24 (1.87) | 2 (1.25) |  |
| BMI, kg/m^2^ |  |  |  |  |  |  |
| <18.5 | 214 (20.22) | 84 (21.88) | 0.18 | 262 (20.44) | 36 (22.50) | 0.52 |
| 18.5-22.9 | 643 (60.78) | 242 (63.02) |  | 784 (61.15) | 101 (63.12) |  |
| 23.0-27.5 | 168 (15.88) | 53 (13.80) |  | 200 (15.60) | 21 (13.13) |  |
| >27.5 | 33 (3.12) | 5 (1.30) |  | 36 (2.81) | 2 (1.25) |  |
| Family background |  |  |  |  |  |  |
| Urban | 744 (70.32) | 285 (74.22) | 0.17 | 916 (71.45) | 113 (70.62) | 0.90 |
| Rural | 314 (29.68) | 99 (25.78) |  | 366 (28.55) | 47 (29.38) |  |
| Being the only child |  |  |  |  |  |  |
| Yes | 639 (60.40) | 227 (59.11) | 0.70 | 777 (60.61) | 89 (55.62) | 0.26 |
| No | 419 (39.60) | 157 (40.89) |  | 505 (39.39) | 71 (44.38) |  |
| Being a left-behind child |  |  |  |  |  |  |
| Yes | 130 (12.29) | 56 (14.58) | 0.29 | 161 (12.56) | 25 (15.62) | 0.33 |
| No | 928 (87.71) | 328 (85.42) |  | 1121 (87.44) | 135 (84.38) |  |
| Paternal educational level |  |  |  |  |  |  |
| Primary school | 117 (11.06) | 43 (11.20) | 0.17 | 139 (10.84) | 21 (13.12) | 0.21 |
| Middle school | 473 (44.71) | 151 (39.32) |  | 565 (44.07) | 59 (36.88) |  |
| College and above | 468 (44.23) | 190 (49.48) |  | 578 (45.09) | 80 (50.00) |  |
| Paternal occupation |  |  |  |  |  |  |
| White collar | 427 (40.36) | 172 (44.79) | 0.57 | 531 (41.42) | 68 (42.50) | 0.16 |
| Blue collar | 162 (15.31) | 51 (13.28) |  | 197 (15.37) | 16 (10.00) |  |
| Farmers | 142 (13.42) | 52 (13.54) |  | 165 (12.87) | 29 (18.13) |  |
| Self-employed | 183 (17.30) | 58 (15.10) |  | 212 (16.54) | 29 (18.13) |  |
| Other | 144 (13.61) | 51 (13.28) |  | 177 (13.81) | 18 (11.25) |  |
| Maternal educational level |  |  |  |  |  |  |
| Primary school | 183 (17.30) | 64 (16.67) | 0.12 | 212 (16.54) | 35 (21.88) | 0.14 |
| Middle school | 509 (48.11) | 165 (42.97) |  | 609 (47.50) | 65 (40.63) |  |
| College and above | 366 (34.59) | 155 (40.36) |  | 461 (35.96) | 60 (37.50) |  |
| Maternal occupation |  |  |  |  |  |  |
| White collar | 345 (32.61) | 149 (38.80) | 0.26 | 435 (33.93) | 59 (36.88) | 0.20 |
| Blue collar | 161 (15.22) | 48 (12.50) |  | 196 (15.29) | 13 (8.13) |  |
| Farmers | 158 (14.93) | 54 (14.06) |  | 188 (14.66) | 24 (15.00) |  |
| Self-employed | 155 (14.65) | 53 (13.80) |  | 182 (14.20) | 26 (16.25) |  |
| Other | 239 (22.59) | 80 (20.83) |  | 281 (12.92) | 38 (23.75) |  |
| Baseline depression ^c^ |  |  |  |  |  |  |
| High | 148 (13.99) | 131 (34.11) | <0.01 | 216 (16.85) | 63 (39.38) | <0.01 |
| Low | 877 (82.89) | 233 (60.68) |  | 1021 (79.64) | 89 (55.63) |  |
| Unknown | 33 (3.12) | 20 (5.21) |  | 45 (3.51) | 8 (5.00) |  |
| Baseline anxiety ^c^ |  |  |  |  |  |  |
| High | 258 (24.39) | 180 (46.88) | <0.01 | 363 (28.32) | 75 (46.88) | <0.01 |
| Low | 767 (72.50) | 184 (47.92) |  | 874 (68.17) | 77 (48.13) |  |
| Unknown | 33 (3.12) | 20 (5.21) |  | 45 (3.51) | 8 (5.00) |  |
| Baseline stress ^c^ |  |  |  |  |  |  |
| High | 108 (10.21) | 107 (27.86) | <0.01 | 168 (13.10) | 47 (29.38) | <0.01 |
| Low | 917 (86.87) | 257 (66.93) |  | 1069 (83.39) | 105 (65.62) |  |
| Unknown | 33 (3.12) | 20 (5.21) |  | 45 (3.51) | 8 (5.00) |  |

BMI, body mass index; IES-R, The Impact of Event Scale-Revised; K6, Kessler 6-item Psychological Distress Scale; N, number; SD, standard deviation.

^a^ Psychological distress using the Kessler 6-item Psychological Distress Scale (K6).

^b^ Acute stress reaction using the Impact of Event Scale-Revised (IES-R).

^c^ Baseline depression, anxiety, and stress were assessed using the Depression Anxiety Stress Scale-21 items in October 2019.

**S3. Supplementary Tables**

**Table S3.** **Health professional students’ perspectives on the COVID-19 outbreak in China.**

| Concerns (n=1431) | No. (%) |
| --- | --- |
| Being infected by COVID-19 | 966 (67.51) |
| Family members or friends being infected by COVID-19 | 950 (66.39) |
| Academic performance | 747 (52.20) |
| Physical health condition | 425 (29.70) |
| Social life and activities | 409 (28.58) |
| Personal or family financial situation | 223 (15.58) |
| Mental health | 213 (14.88) |
| Travel plans | 130 (9.08) |
| Others (e.g., insufficient daily supplies, the outbreak situation, or home quarantine) | 23 (1.61) |
| Needs (n=1442) | No. (%) |
| Personal protective equipment | 1337 (92.72) |
| Social insurance | 1266 (87.79) |
| Salary incentives | 978 (67.82) |
| Clinical practice guidance | 962 (66.71) |
| Professional track record | 882 (61.17) |
| Others (e.g., reasonable work shift, psychological support, or department support) | 11 (0.76) |
| Career choice for the future being affected (n=1442) | No. (%) |
| Yes | 276 (19.14) |
| No | 638 (44.87) |
| Not clear | 528 (37.13) |
| Future career choice (n=1442) | No. (%) |
| Healthcare worker | 808 (56.03) |
| Medicine-related, but not bedside | 448 (31.07) |
| Outside of medicine | 37 (2.57) |
| Indeterminate | 149 (10.33) |
